# Antibacterial Potential of *Limosilactobacillus fermentum* YTPP05 Against Methicillin-Resistant *Staphylococcus aureus*

**DOI:** 10.3390/foods15081431

**Published:** 2026-04-20

**Authors:** Pimnipa Pornjirawittayakul, Peerapat Krittanan, Kittipot Sirichaiwetchakoon, Surachat Buddhisa, Nattaphol Prakobkaew, Ismini Nakouti, Glyn Hobbs, Churat Weeraphan, Htun Htun Win, Benjawan Dunkhunthod, Yothin Teethaisong

**Affiliations:** 1 Department of Medical Sciences, Faculty of Allied Health Sciences, Burapha University, Chon Buri 20131, Thailand; pimnipa.work@gmail.com (P.P.); peerapat.kr@go.buu.ac.th (P.K.); d6200633@g.sut.ac.th (H.H.W.); 2 Division of Pharmacology and Biopharmaceutical Sciences, Faculty of Pharmaceutical Sciences, Burapha University, Chon Buri 20131, Thailand; kittipot.si@go.buu.ac.th; 3 Department of Medical Technology, Faculty of Allied Health Sciences, Burapha University, Chon Buri 20131, Thailand; surachat.bu@go.buu.ac.th (S.B.); nattaphol@go.buu.ac.th (N.P.); 4 Centre for Natural Products Discovery (CNPD), School of Pharmacy and Biomolecular Sciences, Liverpool John Moores University, Liverpool L3 3AF, UK; i.nakouti@ljmu.ac.uk (I.N.); g.hobbs@ljmu.ac.uk (G.H.); 5 Laboratory of Biochemistry, Chulabhorn Research Institute, Bangkok 10210, Thailand; churat@cri.or.th; 6 Thai Tradition Medicine Program, Faculty of Nursing and Allied Health Sciences, Phetchaburi Rajabhat University, Phetchaburi 76000, Thailand; benjawan.dun@mail.pbru.ac.th

**Keywords:** MRSA, *S. aureus*, antibiotics, cell free supernatant, pickled radish

## Abstract

Lactic Acid Bacteria (LAB)-derived antimicrobial compounds are recognized as a promising source of novel antimicrobial agents, particularly for the treatment of Methicillin-Resistant *Staphylococcus aureus* (MRSA), where the mode of action and associated cellular effects remain largely unexplored. This study aims to evaluate antibacterial activity of *Limosilactobacillus fermentum* YTPP05 isolated from pickled radish against MRSA. Upon the initial antibacterial evaluations, it was found that strain YTPP05 inhibited the growth of MRSA isolates. Multiplex PCR identified multiple resistance genes in our MRSA strains, including *mecA*, *blaZ*, and *aacA* genes, aligning with antibacterial susceptibility profiles determined by the disc diffusion assay. An agar overlay assay showed that YTPP05 possessed antibacterial potential, with the largest inhibition zone diameters of 40.83 ± 8.43 mm, while the inhibition zones of the Cell Free Supernatant (CFS) of YTPP05 by an agar well diffusion were 27.16 ± 2.93 mm against the MRSA isolates. The minimum inhibitory concentration and minimum bactericidal concentration of YTPP05-derived CFS were 125 mg/mL. Scanning Electron Microscopy (SEM) demonstrated YTPP05 extracts caused cell membrane disruption, bubble-like protrusion, and cell lysis. Collectively, this study highlights the anti-MRSA potential of YTPP05 as an alternative antimicrobial agent for combating MRSA infections.

## 1. Introduction

*Staphylococcus aureus* (*S. aureus*) can be found ubiquitously on human skin, respiratory mucosa, nasal cavities, and as a food-borne pathogen on contaminated foods [1]. *S. aureus* is one of the pathogens that falls within the ESKAPE group of high-risk pathogens in the development of multidrug resistance (MDR), comprising *Enterococcus faecium*, *Staphylococcus aureus*, *Klebsiella pneumoniae*, *Acinetobacter baumannii*, *Pseudomonas aeruginosa*, and *Enterobacter* spp. [2].

Methicillin-Resistant *S. aureus* (MRSA) infections are prevalent in public healthcare settings. Due to the rising incidence of MRSA infections, it has become a major cause of various human diseases and poses a significant public health risk [3]. MRSA infections range from mild to severe, causing a broad range of diseases from minor skin infections to serious invasive infections affecting multiple body parts and organs [4]. MRSA has been classified by the World Health Organization (WHO) in the “high-priority” pathogen category, highlighting the urgent need for continuous research and development of effective antimicrobial strategies to control rising resistance and widespread transmission [5]. MRSA can be additionally classified into Hospital-Acquired MRSA (HA-MRSA) and Community-Acquired MRSA (CA-MRSA) based on their infection settings. HA-MRSA is commonly considered as a nosocomial infection that transmits among patients, health professionals, and facilities [3], while CA-MRSA can be transmitted among healthy individuals across all age groups in the community settings. Treatment of MRSA infections is considerably challenging [6,7,8].

MRSA confers resistance to penicillin antibiotics by the production of additional Penicillin-Binding Protein 2a (PBP2a), β-lactamase, enhanced efflux pump activity, biofilm formation, and alterations in cell wall function [9]. The PBP2a protein encoded by the *mecA* gene is a predominant resistant mechanism that is responsible for methicillin resistance due to its low affinity for β-lactam antibiotics. In addition to PBP2a production, alterations in cell wall structure and an increase in the thickness of the peptidoglycan layer further reduces the susceptibility to antibiotics. Furthermore, increased activity of the efflux pump systems contributes to MRSA resistance by actively expelling antibiotics from the cell, thereby reducing intracellular drug concentrations [10].

Given these resistance mechanisms and the increasing prevalence of MRSA, the limitations of and reduction in effective conventional antibiotics are evident, highlighting the urgent need for alternative anti-MRSA agents. Several studies have identified and reported that Lactic Acid Bacteria (LAB) isolated from fermented foods show a broad spectrum of antimicrobial activity toward *S. aureus*, *Listeria monocytogenes*, *Escherichia coli*, and *Salmonella* spp. [11,12,13]. Their antimicrobial activities are mediated by antimicrobial peptides (AMPs), organic acids, hydrogen peroxides, and bacteriocins that can inhibit the growth of bacteria through the acid environment, cellular damage, and predominantly membrane-targeting mechanisms [14]. Unlike conventional antibiotics, secreted metabolites from LAB may act synergistically with the antibiotics to resurrect the antibacterial activity, potentially mitigating MRSA resistance mechanisms by enhancing antibiotic efficacy, interfering with efflux pump function, and inhibiting biofilm formation [15,16].

Fermented vegetables harbor diverse LAB populations and represent a rich source of strains with potential anti-MRSA activity [17]. Recent reclassification of lactobacilli based on whole genome studies resulted in the description of 25 genera [18]. *Limosilactobacillus fermentum* (*L. fermentum*) is recognized as a probiotic strain and its direct pathogen antagonism is also widely reported [19]. For instance, *L. fermentum* 3872 produced the bacteriocins BLF3872, which induced the *S. aureus* damage and caused the leakage of the cellular components [20]. Purified metabolic extract of *L. fermentum* KAU0021 exhibited the antibacterial and anti-biofilm properties at sub-MIC concentration in combating *Candida albicans* and *S. aureus* in polymicrobial infections [21]. It has also been reported that isolated *L. fermentum* TCUESC01 inhibited the growth of *S. aureus* and decreased biofilm thickness nearly fivefold [22].

However, the strain-specific nature of LABs on anti-MRSA activity remains poorly understood. Although *L. fermentum* has been isolated from various fermented vegetables, its inhibitory effects against MRSA and underlying cellular effects have not been fully elucidated. Hence, this study aims to evaluate the anti-MRSA potential of the isolated LAB strains from the fermented radish, while investigating their effects on ultrastructural characteristics of *S. aureus*. In addition, we also investigated the antibacterial potency of Cell Free Supernatant (CFS) of *L. fermentum* YTPP05 against MRSA isolates. The possible antibacterial mechanism of action was elucidated by Scanning Electron Microscope (SEM).

## 2. Materials and Methods

### 2.1. Materials

Five isolates of MRSA used in this study included *S. aureus* DMST 20646, DMST 4738, DMST 20649, DMST 20651, and DMST 20652, were obtained from the Department of Medical Sciences, Ministry of Public Health, Nonthaburi, Thailand. A reference strain, *S. aureus* ATCC 29213, was obtained from the American Type Culture Collection (ATCC; Gaithersburg, MD, USA). All bacterial strains were handled and transferred in accordance with standard biosafety guidelines and institutional regulations.

Mueller–Hinton broth (MHB), Mueller–Hinton agar (MHA), and de Man, Rogosa and Sharpe (MRS) medium were purchased from HiMedia (Mumbai, India) and prepared using distilled water according to the manufacturer’s instructions. Commercial antibiotic discs, including ampicillin, amoxicillin, cloxacillin, vancomycin, gentamicin, and tetracycline, were obtained from Oxoid (Hampshire, England, UK) and used for antibiotic susceptibility testing. Standard nisin was acquired from Sigma-Aldrich (Singapore Science Park II, Singapore) and used as a reference antimicrobial agent for the SEM study.

### 2.2. Multiplex PCR

Multiplex PCR analysis was performed to genotypically detect MRSA-harboring antibiotic resistance genes, including *mecA*, *blaZ*, and *aacA*, while the *SAU* (*S. aureus*-specific gene) was employed as an internal control, in the following bacterial strains: MRSA DMST 20646, DMST 4738, DMST 20649, DMST 20651, and DMST 20652. A reference strain of *S. aureus* ATCC 29213 was used as a non-antibiotic-resistant control strain. Primer sequences used in the present study were as described in previous studies [23,24]. Overnight grown bacterial cultures were collected for genomic DNA using commercially available bacterial DNA extraction kits (Vivantis, Selangor, Malaysia). Multiplex PCR reactions were executed in a total volume of 25 µL reaction using GoTaq^®^ Green Master Mix (Promega Cooperation, Madison, WI, USA), 0.5 µM each of primers, 100 ng of genomic DNA template, and DNase free water. PCR amplification was carried out using a Bio-Rad T100 thermal cycler (Hercules, CA, USA) with the following cycling conditions: initial denaturation at 95 °C for 5 min, followed by 30 cycles of 95 °C for 30 s, 60 °C for 30 s, and 72 °C for 1 min, and a final extension at 72 °C for 5 min. PCR products were separated and analyzed with the 1.5% agarose gel electrophoresis comprising SYBR Safe DNA staining dye (Invitrogen, Carlsbad, CA, USA) at 100 V for 45 min. The amplified bands were visualized using a ChemiDoc Touch Imaging System (Bio-Rad, Hercules, CA, USA) [25].

### 2.3. Antibiotic Susceptibility Profiles of MRSA Using a Disk Diffusion Assay

Antibiotic susceptibility of MRSA strains was evaluated using the disk diffusion method according to the standard guideline described by the CLSI (Clinical Laboratory Standards Institute) [26]. Briefly, overnight cultures were pelleted and suspended in 0.9% normal saline solution and adjusted to a 0.5 McFarland standard equivalent. Subsequently, the bacterial suspensions were spread evenly onto the Mueller–Hinton agar (MHA) plates creating a confluent bacterial lawn. Antibiotic discs (10 µg ampicillin, 5 µg amoxicillin, 5 µg cloxacillin, 30 µg vancomycin, 10 µg gentamicin, and 30 µg tetracycline) were aseptically placed onto the inoculated agar surface. Following incubation at 37 °C for 24 h, the inhibition zone surrounding each antibiotic disc was measured [27]. Antibiotic susceptibility was interpreted based on the CLSI breakpoints where applicable.

### 2.4. Isolation and Identification of LAB from Pickled Radish

Pickled white radish samples were obtained from local markets in Chon Buri Province, Thailand. Isolation of LAB was performed according to previously described methods with slight modifications [13]. Briefly, the sample was chopped into small (1–4 mm) cubes and homogenized into sterile normal saline, serially diluted, and 100 µL of the appropriate dilution were evenly spread onto the LAB-specific MRS agar supplemented with 1% CaCO_3_. Following this, the plates were incubated at 37 °C for 48 h. The distinct colonies were chosen based on their unique LAB morphologies and screened by Gram staining.

The isolate having the highest inhibition zone diameter was chosen for species identification by 16S rRNA-specific PCR amplification using universal primers 27F and 1492R. The PCR product was purified and sequenced. The nucleotide sequences were subjected to the BLASTn tool at the National Center for Biotechnology Information (NCBI) website (https://blast.ncbi.nlm.nih.gov/Blast.cgi, last accessed [06 Nov 2025]). The percent identity is described in Section 3.

### 2.5. Agar Overlay Assay and Agar Diffusion Assay

*L. fermentum* YTPP05 showed the highest antibacterial activity, and its activity against MRSA isolates was further confirmed by an agar overlay assay. Briefly, a single colony of *L. fermentum* YTPP05 was selected to culture in the MRS broth at 37 °C for 48 h. The optical density of the bacterial suspension was spectrophotometrically adjusted to 0.1 at a wavelength of 600 nm, and 5 µL of the suspension was spotted onto MRS agar plates. Subsequently, the plates were incubated at 37 °C for 24 h to allow colony development and antibacterial compound production. After incubation, the plates were overlaid with 0.7% (*w*/*v*) soft Mueller–Hinton agar inoculated with 0.5 McFarland standard adjusted *S. aureus* strains (approximately 1.5 × 10^8^ CFU/mL). The overlaid plates were incubated at 37 °C for 24 h; the diameters of the inhibition zones surrounding the LAB colonies were measured.

For the agar well diffusion assay, CFS of YTPP05 was prepared from a 48 h grown culture with cells removed by centrifugation at 6000 rpm. The supernatant was collected and lyophilized to a dry powder, which was subsequently reconstituted in sterile distilled water to achieve a final concentration of 500 mg/mL. To distinguish the antibacterial effect on antimicrobial peptides from organic acids, the antibacterial activity of neutralized CFS (pH 7) was assessed by an agar well diffusion assay. Antibacterial activity was assessed from the overnight cultures of *S. aureus* strains, which were adjusted to a 0.5 McFarland standard and uniformly spread onto MHA using a sterile cotton swab. Wells were aseptically punched using a cork borer, and 40 µL of the reconstituted CFS was added to each well. After the plates were incubated at 37 °C for 24 h, the inhibition zone diameters were measured [28].

### 2.6. Antibacterial Activity Evaluation by MIC and MBC

Determination of MIC and MBC values provided a quantitative insight into the potency and susceptibility of the antimicrobial agents. Based on the MIC and MBC results, bacteriostatic and bactericidal effects could be distinguished. The lowest MIC values demonstrate the greater antibacterial potency that can inhibit the growth whereas the lowest MBC values indicate the concentration that can kill the bacteria. The ratio of MBC/MIC is less than 4 (MBC/MIC ≤ 4) and it is considered as bactericidal whereas bacteriostatic is classified when the MBC/MIC ratio is greater than 4 (MBC/MIC > 4) [29].

The MIC of the YTPP05-derived CFS against MRSA strains was determined using the microdilution method in accordance with the CLSI guidelines [30]. The CFS of YTPP05 was collected from an overnight grown culture and prepared at the required concentrations. For the MIC values, overnight cultures of *S. aureus* were harvested, washed, and adjusted to a 0.5 McFarland standard. The bacterial suspension was further diluted to obtain a final concentration of 5 × 10^6^ CFU/mL. Each well contained 200 µL consisting of 20 µL of bacterial suspension added to 180 µL of cation-adjusted Mueller–Hinton broth (MHB) with the addition of serially diluted CFS of YTPP05. The wells containing broth without antibacterial agents served as a negative and growth control. The plates were incubated at 37 °C for a time range of 18–24 h and bacterial growth was assessed using a microplate reader at a wavelength of 600 nm. The MIC value was determined as the lowest concentration inhibiting the growth of bacteria compared to the untreated growth control.

MBC determination was performed with the 100 µL aliquots that were taken from the wells showing no visible growth at or above the MIC. Then, the aliquots were spread onto Mueller–Hinton agar and incubated at 37 °C for 24 h. MBC values were the lowest concentration that showed no growing bacterial colonies [31].

### 2.7. SEM Analysis

For the SEM analysis, the samples were prepared with the minor modification of the method reported by a previous study [32]. In brief, nisin and CFS of YTPP05 were added to the log-phase-grown *S. aureus*. After 4 h of incubation, bacteria were collected by centrifugation at 6000 rpm. Subsequently, bacterial pellets were collected and fixed with 2.5% (*w*/*v*) glutaraldehyde under cold conditions overnight. Samples were washed with 0.1 M phosphate-buffered saline (pH 7.2) 3 times prior to post-fixation with 1% osmium tetroxide in PBS buffer for 2 h. Samples were washed 3 times and dehydrated using a graded acetone series (20%, 40%, 60%, 80%, and twice at 100%). The cells were transferred to a glass slide and air-dried. Prior to SEM analysis, samples were attached to the stubs with carbon tape and gold coated.

### 2.8. Statistical Analysis

All data from at least three independent experiments were reported as mean ± Standard Deviation (SD). One-way analysis of variance (ANOVA) and the Tukey post hoc test were used for the analysis of statistical differences, with *p* < 0.05. Data analysis was carried out using GraphPad Prism (Version 10, GraphPad Software, San Diego, CA, USA).

## 3. Results and Discussion

### 3.1. Detection of Antibiotic Resistance Genes Using Multiplex PCR

**Figure 1:**
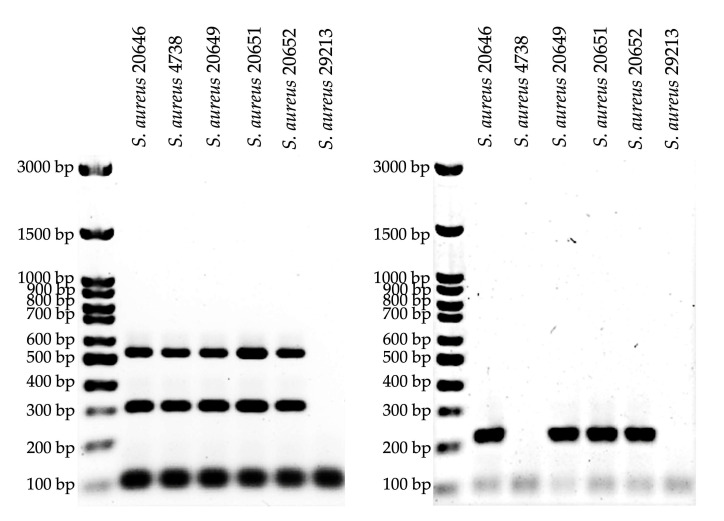
Multiplex PCR for the detection of antibiotic-resistant encoding genes (*mecA*, *blaZ*, *aacA*, and *SAU*). The 1.5% agarose gel was used for PCR amplicons analysis.

The rise of MRSA is primarily mediated by *mecA* and *blaZ* genes and poses a serious threat to public health globally [33]. Multiplex PCR allows for the rapid identification of several antibacterial resistance genes in one reaction [23]. The multiplex PCR assay identified *mecA*-encoded PBP2a and *blaZ*-encoded penicillinase in all five MRSA strains. In addition, *aacA*-encoded aminoglycoside-modifying enzymes were detected in MRSA DMST 20646, DMST 20649, DMST 20651, and DMST 20652 (Figure 1). No resistant genes were detected in the reference strain.

The findings are consistent with previous studies reporting that *mecA* and *blaZ* genes are the predominant resistance genes in MRSA [34]. In addition, aminoglycoside resistance encoded by the *aacA* gene was detected in four DMST isolates. These results underscore the co-occurrence of multiple resistance genes in individual strains, confirming the presence of multidrug resistance [35].

### 3.2. Antibiotic Susceptibility Profile of MRSA Strains

Antibiotic susceptibility profiling is a fundamental approach for evaluating the resistance behaviors of microorganisms. The disk diffusion method is among the most commonly used techniques to assess susceptibility to different antimicrobial classes. Inhibition zone diameters represent the strain dependent efficacy and resistance levels.

**Figure 2:**
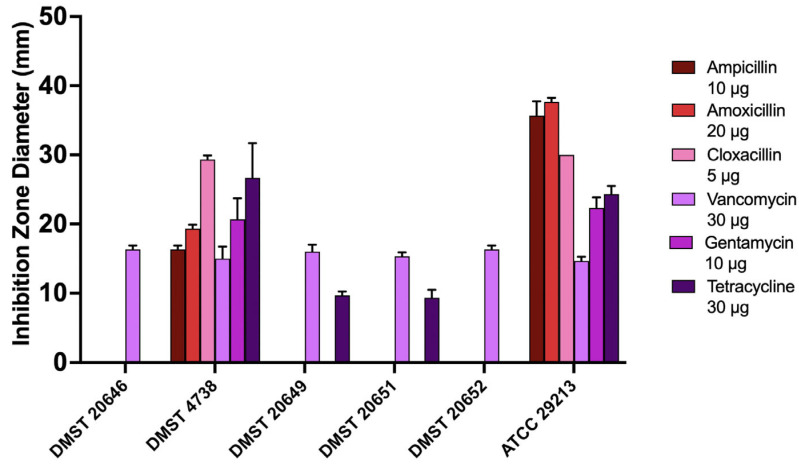
Antibiotic susceptibility profile of *S. aureus* isolates. The values are expressed as diameter in millimeter (mm) of mean ± standard deviation (*n* = 3).

Figure 2 demonstrates the variable inhibition zone diameters and expresses susceptibility patterns across the tested strains. Ampicillin, amoxicillin, cloxacillin, and gentamycin significantly reduced susceptibility in four DMST strains, while differences in the response to vancomycin and tetracycline were also noted.

In contrast, *S. aureus* ATCC 29213 displayed the largest inhibition zone diameter, with amoxicillin (37.6 ± 0.5 mm) and ampicillin (35.6 ± 2 mm) showing the highest activity. The smaller inhibition zone diameters were observed for the remaining antibiotics. These findings align with the previous studies describing variable antibiotic susceptibility among clinical and reference *S. aureus* strains [36]. The absence of resistance genes in *S. aureus* ATCC 29213, together with its higher susceptibility, supports the multiplex PCR assay results and validates the use of these strains for evaluating the antibacterial activity of LAB-derived compounds.

### 3.3. Isolation, Identification, and Morphological Analysis of Antibacterial-Producing LAB from Pickled Radish

**Figure 3:**
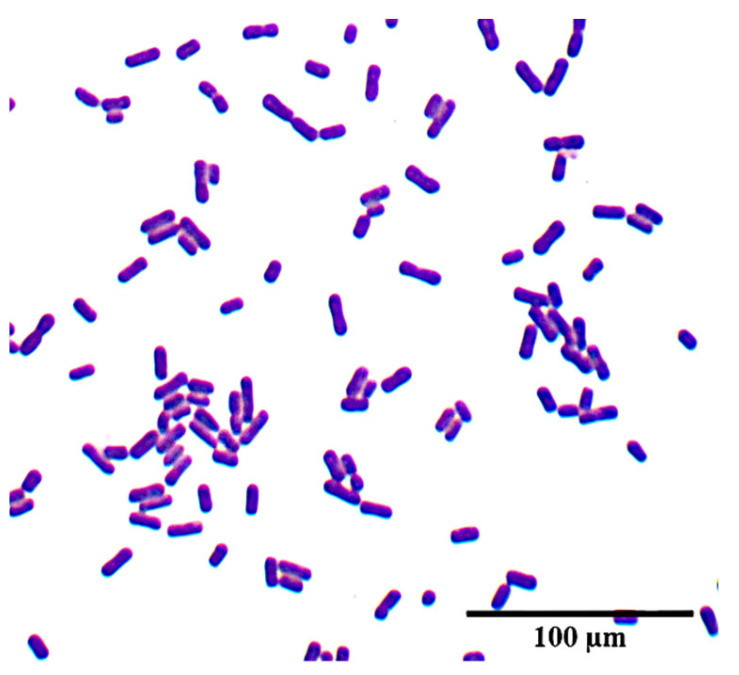
Microscopic morphologies of isolated strain *L. fermentum* YTPP05.

Screening of bacteria that produced antibacterial activity against MRSA is crucial for the selection of promising candidates for subsequent antimicrobial evaluation. Therefore, LAB were selectively isolated from pickled radish from the local market. Based on their general colony morphologies, such as shape, color, size, and convexity, the *L. fermentum* YTPP05 strain was found to show the highest antibacterial potential against MRSA and *S. aureus* isolates. Gram staining confirmed that the isolates were Gram-positive rods, as illustrated in Figure 3.

Identification based on 16S rRNA gene sequencing and BLAST analysis revealed 100% sequence similarity with *L. fermentum* SL5-1 (GenBank accession number MN435802.1).

Fermented foods are a rich source of lactobacillus species. *L. fermentum* was shown and confirmed as probiotic as well as having typical antimicrobial properties, such as the production of bacteriocins [37]. A previous study reported that antimicrobial peptides (AMPs) from *L. fermentum* RC-14 are shown to be effective in inhibiting the growth of *S. aureus* [38]. Furthermore, class III bacteriocin-producing *L. fermentum* 3872 isolated from the milk of breastfeeding women possessed antibacterial properties against antibiotic-resistant *S. aureus* [20]. Overall, the isolated strain that had the strongest inhibition zone diameter could be a promising candidate for inhibiting the growth of MRSA strains.

### 3.4. Agar Overlay and Agar Well Diffusion Assays

A total of five isolates of MRSA (DMST 20646, DMST 4738, DMST 20649, DMST 20651, and DMST 20652) and one reference strain (ATCC 29213) were examined to determine the antimicrobial properties of *L. fermentum* YTPP05. Among the antibacterial activity assays, the agar overlay was applied to determine the antibacterial activity of *L. fermentum* YTPP05 grown on MRS agar and overlaid with soft agar containing the bacteria. The basic principle of the agar overlay assay is a double-layer technique allowing the antimicrobial agents to diffuse more and evenly from the bottom layers into the top agar layer [39]. Whereas the agar well diffusion assay was employed for evaluating the antibacterial potency of CFS.

**Figure 4:**
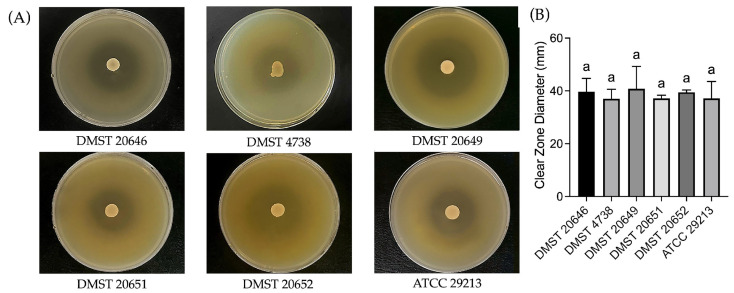
Antibacterial activity of *L. fermentum* YTPP05 on (**A**) agar overlay assay and (**B**) inhibition zone diameters against the *S. aureus* isolates. Data are described as mean ± S.D., one-way ANOVA analysis with Tukey’s multiple comparison test (*p* < 0.05). The same letter “a” represents no significant difference.

The antibacterial activity of *L. fermentum* YTPP05 determined by an agar overlay technique demonstrated the high antibacterial potential of YTPP05 against all tested MRSA and the reference strain, with no significant difference in inhibition zone diameters among bacteria. MRSA DMST 20649 exhibited the largest inhibition zone (40.83 ± 8.43 mm), but the overall inhibition pattern remained consistent. These results indicate that *L. fermentum* YTPP05 exhibits promising antibacterial activity against MRSA strains (Figure 4).

**Figure 5:**
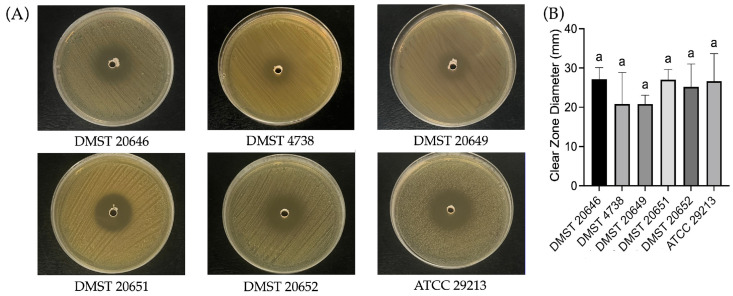
Antibacterial activity of CFS of *L. fermentum* YTPP05 on (**A**) agar well assay and (**B**) inhibition zone diameters against the *S. aureus* isolates. Data are reported as mean ± S.D., one-way ANOVA analysis with Tukey’s multiple comparison test (*p* < 0.05). The same letter “a” represents no significant difference.

The agar well diffusion assay demonstrated the antibacterial activity of the CFS, indicating the YTPP05 secreted antimicrobial compounds. No significant differences were observed among the tested bacteria (Figure 5). DMST 20646 and DMST 20651 demonstrated inhibition zone diameters of 27.16 ± 2.93 mm and 27.00 ± 2.65 mm, respectively. The inhibition patterns obtained from the agar well diffusion assay were consistent with those observed in the agar overlay assay and further supported the potential of the CFS of *L. fermentum* YTPP05 as an antibacterial agent.

**Figure 6:**
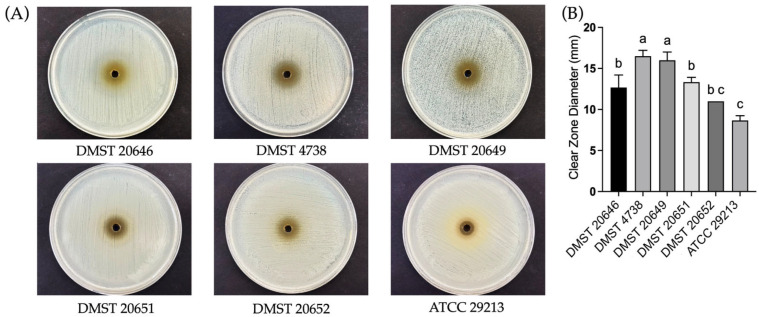
Antibacterial activity of neutralized CFS (pH 7) of *L. fermentum* YTPP05 on (**A**) agar well assay and (**B**) inhibition zone diameters against the *S. aureus* isolates. Data are reported as mean ± S.D., one-way ANOVA analysis with Tukey’s multiple comparison test (*p* < 0.05). The letters “a, b, c” represent a significant difference.

The neutralized CFS of *L. fermentum* YTPP05 exhibited antibacterial activity, but its inhibition zone diameters were significantly lower than the crude CFS (Figure 6), indicating CFS contains antimicrobial peptides. The results from an agar well diffusion suggest that both antimicrobial peptide and organic acids, such as lactic acid and acetic acid, in the CFS play an antimicrobial role against MRSA isolates. Our findings are consistent with a previous study that organic acids produced by LAB had antimicrobial potential [40]. Previous studies of the agar well diffusion analysis of *L. fermentum* LAB-1 have reported the strong inhibitory activity of the CFS of the isolated strain against 10 pathogens, including *S. aureus* [12]. Several studies of the gene annotations of *L. fermentum* revealed the presence of putative peptides such as endolysin A, endolysin, and epidermidin-like peptide. The antimicrobial activity of *L. fermentum* is possibly related to the presence of the aforementioned antimicrobial peptide and organic acids, and this indicates that the organism is a promising candidate as a novel antimicrobial producer [41,42].

### 3.5. MIC and MBC Determinations

The MIC and MBC values of CFS from YTPP05 were assessed against six bacterial isolates of *S. aureus*. The MIC of CFS of YTPP05 was 62.5 mg/mL, while MBC was 125 mg/mL across the tested strains (Table 1).

Previous studies of CFS from LAB isolates reported the MIC value of 50 mg/mL and MBC value of >100 mg/mL regarding *S. aureus* DMST 2928 and demonstrated antibacterial potency [43]. CFS from our isolated strain YTPP05 showed higher MIC and MBC concentrations. These relatively higher concentrations of LAB-derived CFS may be attributed to the complex and heterogeneous nature of antimicrobial components present in LAB-secreted supernatants, as well as culture and pH conditions [44]. The concentration of metabolites in CFS is directly related to bacterial growth conditions, initial inoculum effect, incubation temperature, and pH. Variations in these conditions may significantly affect the contents of antimicrobial compounds [45]. In addition, the individual antimicrobial components within the mixture are often relatively low in concentration, and higher quantities of CFS are typically required to achieve bacteriostatic or bactericidal effects [46]. However, future investigations should focus on the effect of culture conditions, such as culture time, temperature, and pH, on the production of antibacterial compounds and antibacterial potential of YTPP05.

In summary, the higher concentration of CFS that exerts bacteriostatic or bactericidal activity is likely influenced by the total concentration of supernatant and initial cell density of inoculum, which together determine the effective concentration of antimicrobial constituents [47]. Overall, these results are consistent with previous studies and reflect the complex nature of antimicrobial metabolites present in LAB-derived supernatants.

### 3.6. Scanning Electron Microscope (SEM)

LAB are present in various fermented foods derived from both animal and plant-based products. It is well-established that the antibacterial activity of LAB is strain dependent, and their efficacy differs depending on the sources [48,49]. Many studies have investigated and supported the fact that LAB secrete antibacterial substances, including bacteriocins [50] and organic acids [51]. Hence, we observed the antibacterial potential of the strain YTPP05 against *S. aureus*. The ultrastructural characteristics of *S. aureus* were visualized using SEM. *S. aureus* was treated with CFS of YTPP05 at a concentration of 0.5× and 1× MIC of YTPP05-derived CFS with nisin as a positive control agent.

**Figure 7:**
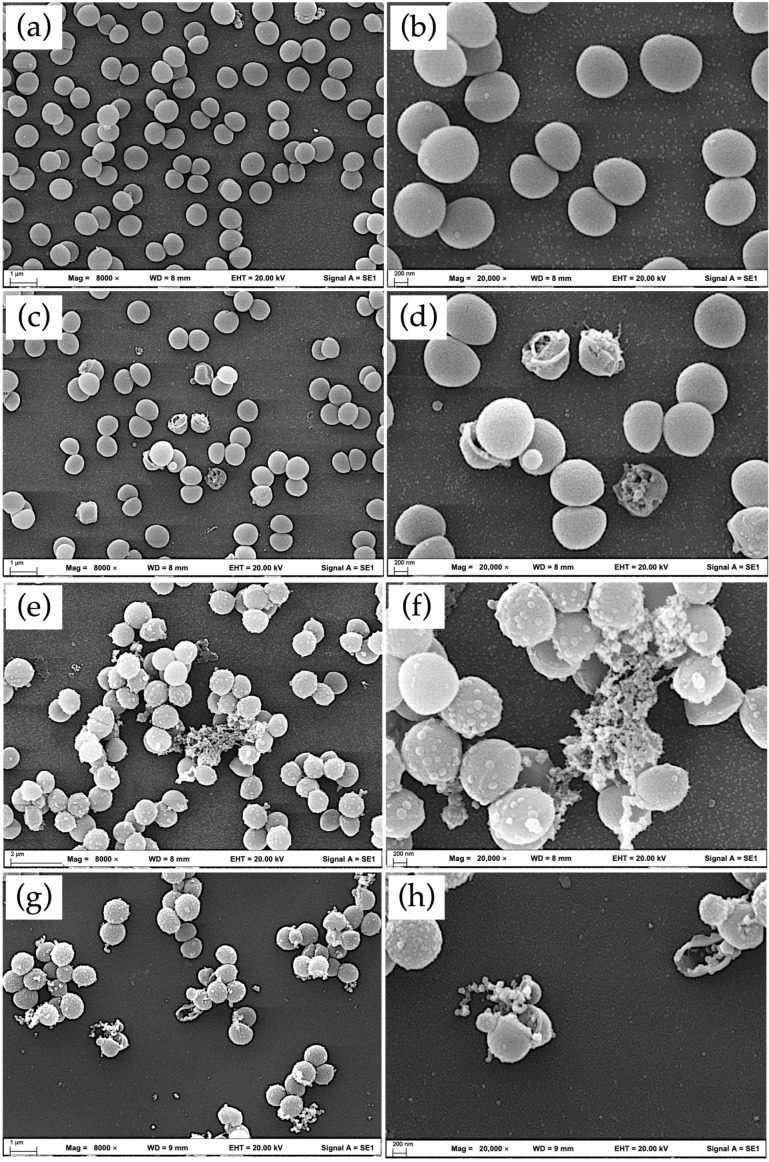
Scanning electron microscopic images of *S. aureus* DMST 20651 control and treated with nisin: (**a**) untreated control—Mag 8000×, (**b**) untreated control—Mag 20,000×, (**c**) nisin—Mag 8000×, (**d**) nisin—Mag 20,000×, (**e**) 1× MIC YTPP05—Mag 8000×, (**f**) 1× MIC YTPP05—Mag 20,000×, (**g**) 0.5× MIC YTPP05—Mag 8000×, and (**h**) 0.5× MIC YTPP05—Mag 20,000×. (Mag—Magnification).

Nisin, a cationic polypeptide produced by *Lactococcus lactis*, at a concentration of 0.5× MIC was used as a positive control due to its antibacterial mechanisms of action by disrupting bacterial cell membrane and interrupting cell wall biosynthesis [52]. It is a highly potent antimicrobial agent against *S. aureus* and has been widely used for treating antibiotic-resistant bacteria. *S. aureus* cells treated with nisin showed pronounced morphological alterations, including cell envelope rupture, collapse, cell lysis, and the presence of cellular debris (Figure 7c,d). Previous Transmission Electron Microscopy (TEM) studies similarly reported nisin-induced membrane damage accompanied by chromosomal DNA damage and cell shrinkage [53]. In the present study, nisin-treated cells also displayed cell lysis, loss of membrane integrity, cell bursting, and reduced cell density compared with the control group. These observations demonstrate that nisin induces extensive cellular damage, whereas untreated cells maintain a normal morphology and structural integrity. Nisin has been reported to inhibit bacterial growth and targets and binds to the lipid II precursor of the cell wall, further disrupting peptidoglycan synthesis and causing leakage of cellular contents [52].

The untreated control group exhibited smooth cell wall surfaces, intact structures, and typical coccoid morphology, with cells evenly separated and arranged in small clusters, indicating well-preserved cell envelope integrity (Figure 7a,b). Following treatment with 1× MIC of YTPP05 CFS, *S. aureus* cells exhibited pronounced surface irregularities, including membrane blebbing, bubble-like protrusions, and localized swelling (Figure 7e,f). These vesicular protrusions were thought to be associated with the formation of membrane-bound structures called mesosomes and are usually formed under stress conditions, particularly when cells are exposed to membrane damaging antimicrobial compounds [54]. Initially, mesosome formation was assumed as an artifact resulting from fixation during microscopy preparation [55].

However, later studies suggested that mesosome formation is related to antibiotic-induced stress, as such structures are often observed in treated bacteria but are less frequently detected in untreated cells [54]. This observation aligns with our finding that the untreated control showed no bubble-like protrusions. Although the specific functions of mesosomes remain unclear, they have been reported to have an association with cellular remodeling under stress conditions. In addition, some studies have suggested that the sizes of mesosomes are proportional to the accumulation of reactive oxygen species, such as hydrogen peroxide, within bacterial cells [56].

Furthermore, cells tended to aggregate, with abnormal protrusions extending toward neighboring cells. Similar surface protrusions and small cell clusters have been reported in previous studies describing the early effects of rhodomyrtone exposure, which has been characterized as a membrane-disrupting agent against *S. aureus* [57].

At 0.5× MIC of CFS of YTPP05, cells formed irregular clusters (Figure 7g,h), and this agrees with the previous report of the growth inhibition of *S. aureus* by the strain *L. plantarum* LR-14 obtained from Sichuan pickles [58]. A remarkable increase in bubble-like protrusions on the cell surface was observed at 0.5× MIC, while these features were slightly reduced with the 1× MIC treatment. Additionally, some cells exhibited complete rupture and progressed to cell lysis. Similar findings were reported in studies from Korea, where treatment with CFS of *L. plantarum* NIBR97 derived from kimchi substantially reduced *S. aureus* populations and caused membrane lysis, likely mediated by antimicrobial peptides such as plantaricin-5 [59].

Membrane damage appeared to be MIC-dependent, with extensive envelope disruption, such as bubble-like protrusions, observed at lower MIC concentrations. In contrast, higher MIC concentrations predominantly induced a loss of membrane integrity and cell death. Organic acids are generally present in LAB-derived CFS, which can reduce the environmental pH and play a crucial role in contributing to antimicrobial activity by disrupting membrane integrity [60]. The pH of CFS used in this study was approximately 6 at a stock concentration of 500 mg/mL, which is within the tolerance range reported for *S. aureus* (pH 4–9) [61]. Moreover, 0.5× and 1× MIC concentrations of CFS (62.5 and 125 mg/mL, respectively) were used for SEM analysis and would be expected to exert less pH influence on the pH of the culture. Therefore, the observed morphological alterations in the SEM results are likely associated with antimicrobial metabolites present in the CFS.

Moreover, it is noteworthy that the antimicrobial activities of CFS and purified bacteriocins are fundamentally different. The positive control agent nisin is a purified and well-characterized lantibiotic bacteriocin. The specific mechanism involves the pore formation in bacterial cell membranes and the inhibition of cell wall biosynthesis [52]. In contrast, CFS derived from LAB may comprise a complex mixture of antibacterial compounds and secondary metabolites secreted during bacterial fermentation. CFS contained several metabolites that are not only bacteriocin but also organic acids, such as lactic acid and acetic acid; hydrogen peroxide; and other antimicrobial compounds [51].

Overall, SEM analysis revealed clear evidence that YTPP05 disrupts the membrane integrity of *S. aureus*. These results demonstrate that antimicrobial substances produced by the YTPP05 strain can effectively inhibit MRSA, highlighting their potential as alternative antimicrobial agents. However, this study did not investigate molecular mechanisms such as antimicrobial peptide identification, KEGG pathway analysis, or whole-genome sequencing. Therefore, further studies focusing on genome-based analyses are required to identify genes involved in the antibacterial mechanisms of YTPP05.

## 4. Conclusions

Taking all the data into account, *L. fermentum* YTPP05 and its CFS exhibited a strong inhibitory effect against MRSA. Multiplex PCR analysis identified *mecA*, *blaZ*, and *aacA* genes in the MRSA isolates, aligning with antibacterial susceptibility testing by the disk diffusion assay. These findings indicate that MRSA isolates used in this study are resistant to multiple antibiotics. Our SEM observation demonstrated and strongly supported the idea that the CFS of *L. fermentum* YTPP05 inhibited MRSA strains through the disruption of the bacterial cell membrane and cell wall. Our *L. fermentum* YTPP05 isolated from pickled white radish could be a promising alternative antibacterial agent to combat pathogenic MRSA strains. However, the identification of antibacterial compounds produced by *L. fermentum* YTPP05, optimization of pH on antibacterial compound production, and efficacy and toxicity in animal and human models should be investigated further.

## Figures and Tables

**Table 1:** MIC and MBC values of YTPP05-derived CFS on *S. aureus* isolates.

Bacterial Isolates	YTPP05 (CFS)	
	MIC (mg/mL)	MBC (mg/mL)
MRSA DMST 20646	125	125
MRSA DMST 4738	125	125
MRSA DMST 20649	125	125
MRSA DMST 20651	125	125
MRSA DMST 20652	125	125
*S. aureus* ATCC 29213 *	62.5	125

* A reference strain for antimicrobial susceptivity testing.

## Data Availability

The original contributions presented in this study are included in the article. Further inquiries can be directed to the corresponding authors.

## Abbreviations

The following abbreviations are used in this manuscript:

CFS	Cell Free Supernatant
MRSA	Methicillin-Resistant *Staphylococcus aureus*
LAB	Lactic Acid Bacteria
PCR	Polymerase Chain Reaction
MIC	Minimum Inhibitory Concentration
MBC	Minimum Bactericidal Concentration
SEM	Scanning Electron Microscope
